# X-Linked Agammaglobulinemia Case with TH Domain Missense Mutation in Bruton Tyrosine Kinase

**DOI:** 10.1007/s10875-020-00962-9

**Published:** 2021-01-27

**Authors:** Nourhen Agrebi, Giusy Gentilcore, Jean-Charles Grivel, Ghroob Alkhayer, Jihad Hassoun, Amel Hassan, Mehdi Adeli, Bernice Lo

**Affiliations:** 1 Research Department, Sidra Medicine, Doha, Qatar; 2 Deep Phenotyping Core, Sidra Medicine, Doha, Qatar; 3 Damascus University Children’s Hospital, Damascus, Syria; 4 AlFayhaa Hospital, Damascus, Syria; 5 Division of Allergy & Immunology, Sidra Medicine, Doha, Qatar

To the Editor:

X-linked agammaglobulinemia (XLA) is a fully penetrant X-linked recessive immunodeficiency disorder, characterized by recurrent bacterial infections, near complete absence of B cells and low immunoglobulin levels. XLA is caused by more than 800 different mutations in the Bruton’s tyrosine kinase (*BTK*) gene, spread over all domains of the BTK protein. So far, only a few variants have been reported to alter the TH (Tec homology domain) and SH3 domains of BTK [1]. In XLA patients diagnosed at an older age (> 6 years), missense mutations that result in BTK protein expression have been found in the various domains of BTK, except the TH domain [2]. Furthermore, the previously reported rare missense substitutions that allowed BTK expression enabled the minute number of CD19^+^ B cells to produce higher than expected amounts of immunoglobulins [2, 3].

Here, we present a case of XLA harboring a missense *BTK* mutation in the TH domain that results in deficient BTK protein expression.

A 3-year-old boy presented with a history of a *Pseudomonas aeruginosa* retropharyngeal abscess at 1 year of age that required surgical incision and drainage, as well as mechanical ventilation and antibiotics that was complicated by pseudomembranous colitis. He had moved to Qatar at 13 months of age where he underwent an immunological evaluation. Relevant laboratory findings showed low IgA and IgM levels but normal IgG and normal T and NK cell numbers coupled to a virtual absence of B cells (0.27%).

Patient had good antibody responses to *Tetanus* and *Diphtheria* but poor response to *Haemophilus influenzae type b* and pneumococcal vaccine (Table 1, Supplemental Data).

Invitae Primary Immunodeficiency Panel sequencing analysis revealed a hemizygous missense variation c.464G>A in exon 6 of *BTK*. The variant is absent from gnomAD and also not found in a published Qatari population dataset [4]. The variant was predicted to be highly deleterious by PolyPhen2 and Mutation-Taster and classified as variant of uncertain/unknown significance (VUS). c.464G>A causes a missense change p.C155Y and has been previously reported in a 31-year-old XLA-patient presenting an increased susceptibility to bacterial infections, decreased serum immunoglobulin levels, and reduced B cells numbers but was never explored for BTK expression [5]. The substitution is located on the highly conserved TH domain (Fig. 1b), known to bind a Zn^2+^ ion which packs against the ß-sheet of the PH domain. Mutation of this site is predicted to result in an unstable protein, leading to undetectable BTK protein [6–8].

**Fig. 1:**
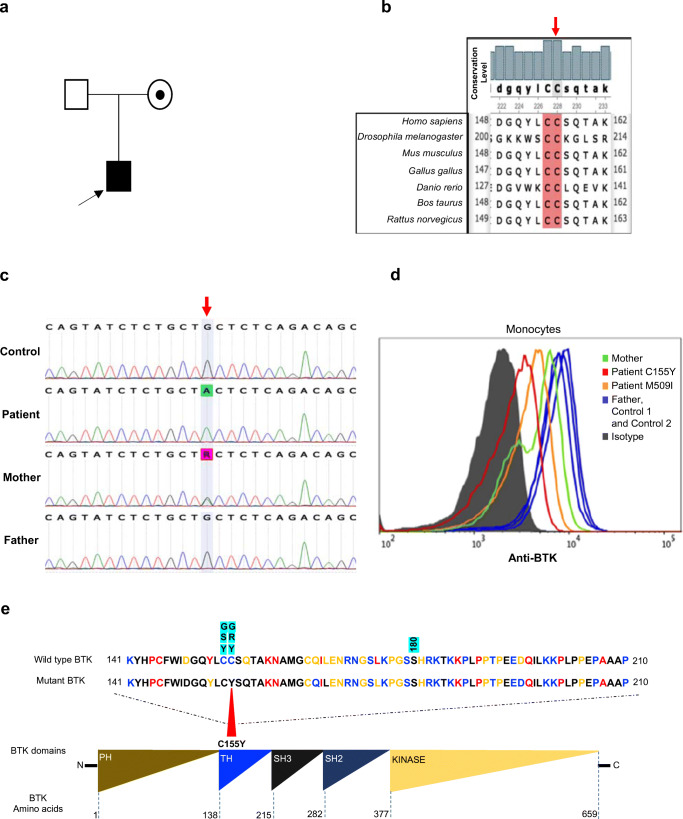
**a** Family pedigree. **b** Multiple protein alignment showing the conservation level of C155 residue in different species (threshold 100%). **c** Sanger sequence analysis of the BTK gene showing the hemizygous (c.464 G>A) mutation. The mother is heterozygous. **d** Flow cytometry assessment of intracellular BTK expression was performed on gated monocytes in the patient (C155Y) and parents. The patient with the M509I BTK mutation was included as a disease control. Normal BTK expression was observed in the father and two healthy controls (control 1 and control 2). Data shown is representative of two independent experiments. **e** Pairwise alignment between wild-type and mutant BTK proteins. Variations discussed in this report have been highlighted with a blue background above the protein sequence. The current case report mutation C155Y is marked with red arrow

BTK protein expression in monocytes of the patient and his parents was analyzed by flow cytometry. Flow cytometric analysis of BTK expression is shown in Fig. 1d. We found that our patient displayed reduced expression of BTK in monocytes. The father’s monocytes present with normal BTK expression, and the mother exhibited a bimodal pattern of monocyte BTK expression, confirming that she is an XLA carrier, which we verified by Sanger sequencing (Fig. 1c and d). In order to explore the degree to which BTK was reduced, we compared the BTK expression of this patient to that of another patient with a known pathogenic M509I missense mutation in the kinase domain of BTK [9]. Interestingly, the C155Y mutation showed a more severe reduction of BTK expression than the M509I mutation, which was previously shown to result in approximately 57% BTK expression (Fig. 1d).

In a previous report, the mutation c.463T>G caused a cysteine to glycine substitution p.C155G presented by a 35-year-old XLA patient with a mild phenotype and a complete absence of B cells [8]. A different publication described an arginine change for the same invariant cysteine p.C155R suggesting its importance in structural integrity and binding of metal cations [10].

The p.C155Y mutation described here is due to a G464 to A alteration that leads to the replacement of the invariant cysteine at codon 155 by Tyrosine (p. Cys155→Tyr), located in the TH domain of BTK protein. A cysteine to serine mutation at position 154 (1 codon before) was reported in a moderately severe XLA patient showing symptoms since the age of 1 year [8]. At this latter position, another cysteine to tyrosine change was reported in a 2-year-old XLA patient showing very low B cells (0.6%) and hypogammaglobulinemia associated with very low BTK expression (1.7%) [11]. As indicated in *BTKbase*, the majority of the missense mutations in the TH domain affect these two highly conserved cysteines C154 and C155 [1]. Of these mutations, half are substitutions to an amino acid bearing a hydroxyl group such as tyrosine and serine, indicating that these changes might harbor a more serious impact on the protein. Interestingly, BTK function has been shown to be negatively regulated when a serine at position 180 of the TH domain is phosphorylated by protein kinase C-β, resulting in impaired membrane localization of BTK [12]. Thus, these mutations might form a phosphorylation cluster (or “hot spot”) that could downregulate the function/expression of BTK and cause an abnormal subcellular localization of BTK protein in the cell cytoplasm. Future studies would be required to investigate this possibility.

Furthermore, residue C155 is predicted to be a potential palmitoylation site as shown by ModPred software (score 0.77). Palmitoylation is a common post-translational modification (PTM) of cysteine residues and plays an important role in the regulation of protein subcellular localization, stability, protein trafficking, and translocation to lipid rafts, among many other protein functions. Although the N-terminal PH domain is involved in mediating binding of BTK to the cell membrane, this association may be strengthened by palmitoylation at the nearby cysteine C155. In fact, a palmitoylated cysteine string has already been reported in TXK, a Tec family member of BTK, providing a similar membrane-binding function as the PH domain [13].

In summary, we report a hemizygous C155Y mutation in *BTK* associated with XLA immune deficiency and demonstrate that this mutation results in highly reduced BTK protein expression. It is tempting to speculate that substitutions of the conserved cysteine C154 or C155 located in the TH domain of BTK protein lead to a more severe impact on BTK protein expression by causing near absence of detectable BTK, compared with other BTK missense variants affecting the TH domain. We also speculate on the functional impact of altered PTMs on the TH domain due to mutation and subsequent low BTK protein detection. Furthermore, we provide data supporting the reclassification of C155Y from VUS to pathogenic. Clearly, a definitive genetic diagnosis will facilitate early diagnosis and treatment of XLA disease, preventing life-threatening complications and worsening clinical outcomes.

## Supplementary Information

ESM 1 (DOCX 19.1 kb)

## Data Availability

All data generated or analysed during this study are included in this article.
