# Supplementary material for: X-Linked Agammaglobulinemia Case with TH Domain Missense Mutation in Bruton Tyrosine Kinase
Source: J Clin Immunol. 2021 Jan 27;41(4):825–8. doi: 10.1007/s10875-020-00962-9 (PMC8068703; doi:10.1007/s10875-020-00962-9)
Supplement: Supplementary file 1 — (DOCX 19.1 kb) [file 10875_2020_962_MOESM1_ESM.docx]

**Supplemental Data**

**TABLE I.** Immunological features of the patient

| **Sex:** Male | |  | |
| --- | --- | --- | --- |
| **Present age (Years):** 3 | |  |  |
| **Age at diagnosis (Months):** 13 | |  |  |
|  | | **Patient Result** | **Normal range** |
| **Lymphocyte phenotypic studies** | | | |
| CD3^+^ (%) | | 93.70 | 54.00-76.00 |
| CD3^+^/CD4^+^ (%) | | 60.40 | 31.00-54.00 |
| CD3^+^/CD8^+^ (%) | | 29.00 | 12.00-28.00 |
| CD19 (%) | | 0.27 | 15.00-39.00 |
| CD3^-^/CD16^+^/CD56^+^ (%) | | 4.90 | 3.00-17.00 |
| CD4:CD8 Ratio | | 2.08 | 1.34–3.04 |
| CD45RA (%) | | 77.30 | 66.3-89.4 |
| CD45RO (%) | | 16.20 | 10.6-33.7 |
| Neutrophils Function | | Normal | - |
| **Serum-specific antibody measurements** | | | |
| Diphtheria Toxoid IgG (IU/mL) | | 0.360 | >0.01^a^ |
| Haemophilus Flu Type B (mg/L) | | <0.11 | >0.15^a^ |
| Pneumococcus Capsular PS (mg/L) | | <3.33 | Positive: > 3.3  Negative: < 3.3 |
| Tetanus Toxoid (IU/mL) | | 0.13 | >0.1^a^ |
| **Immunoglobulin levels** | | | |
| IgG (mg/dL) | At 12 Months of age | 286 | 345.0-1,213.0 |
|  | At 13 Months of age | 477.0 |  |
| IgG Sub 1 (mg/dL) | | 399.0 | 265.0-938.0 |
| IgG Sub 2 (mg/dL) | | 21.2 | 28.0-216.0 |
| IgG Sub 3 (mg/dL) | | 77.4 | 8.7-86.4 |
| IgG Sub 4 (mg/dL) | | 11.6 | 0.9-74.2 |
| IgG (mg/dL) | At 18 Months of age | 406.0 | 345.0-1,213.0 |
| IgG Sub 1 (mg/dL) | | 374.0 | 265.0-938.0 |
| IgG Sub 2 (mg/dL) | | 36.8 | 28.0-216.0 |
| IgG Sub 3 (mg/dL) | | 75.7 | 8.7-86.4 |
| IgG Sub 4 (mg/dL) | | 15.3 | 0.9-74.2 |
| IgG (mg/dL) | At 19 Months of age | 403.0 | 345.0-1,213.0 |
| IgG Sub 1 (mg/dL) | | 379.0 | 265.0-938.0 |
| IgG Sub 2 (mg/dL) | | 36.7 | 28.0-216.0 |
| IgG Sub 3 (mg/dL) | | 64.2 | 8.7-86.4 |
| IgG Sub 4 (mg/dL) | | 12.9 | 0.9-74.2 |
| IgG (mg/dL) | At 21 Months of age | 370.0 | 345.0-1,213.0 |
| IgG Sub 1 (mg/dL) | | 344.0 | 265.0-938.0 |
| IgG Sub 2 (mg/dL) | | 23.4 | 28.0-216.0 |
| IgG Sub 3 (mg/dL) | | 44.1 | 8.7-86.4 |
| IgG Sub 4 (mg/dL) | | 6.1 | 0.9-74.2 |
| IgG (mg/dL) | At 22 Months of age | 346.0 | 345.0-1,213.0 |
| IgG Sub 1 (mg/dL) | | 331.0 | 265.0-938.0 |
| IgG Sub 2 (mg/dL) | | 23.3 | 28.0-216.0 |
| IgG Sub 3 (mg/dL) | | 48.4 | 8.7-86.4 |
| IgG Sub 4 (mg/dL) | | 6.4 | 0.9-74.2 |
| IgA (mg/dL) | At 12 Months of age | 13.9 | 14.0-106.0 |
|  | At 13 Months of age | <5.8 |  |
|  | At 18 Months of age | 8.0 |  |
|  | At 19 Months of age | <6.4 |  |
|  | At 21 Months of age | 6.7 |  |
|  | At 22 Months of age | 7.0 |  |
| IgM (mg/dL) | At 12 Months of age | 34 | 43.0-173.0 |
|  | At 13 Months of age | 25.9 |  |
|  | At 18 Months of age | 9.8 |  |
|  | At 19 Months of age | 6.3 |  |
|  | At 21 Months of age | 13.2 |  |
|  | At 22 Months of age | 10.1 |  |
| Total IgE (mg/dL) | At 18 Months of age | 94.70 | 45-76 |
|  | At 21 Months of age | 89.10 |  |
| **Complement component analysis** | | | |
| C3 (mg/dL) | | 101.0 | 84-174 |
| C4 (mg/dL) | | 34.1 | 12-40 |

^a^ Minimum concentration considered protective against infection
